# Timeline of Achieving a Differentiated RPE Phenotype and Specialised Metabolism of ARPE-19 Cells Under Physiologically Relevant-Inducing Culture Conditions

**DOI:** 10.3390/cells15201846

**Published:** 2026-10-12

**Authors:** Ioan V. Matei, Daniel Grimes, Gunticha Suwanmanee, James Bagnall, David G. Spiller, Luminita Paraoan

**Affiliations:** 1 Ocular Molecular Biology and Mechanisms of Disease Group, School of Biological and Chemical Sciences, Manchester Metropolitan University, Manchester M1 5GD, UK; 2 Department of Biology, Edge Hill University, Ormskirk L39 4QP, UK; 3 Faculty of Biology, Medicine and Health, University of Manchester, Manchester M13 9PL, UK

**Keywords:** ARPE-19, RPE, differentiation, mitochondria

## Abstract

**Highlights:**

**What are the main findings?**

• Our findings support the notion that most important processes can be successfully interrogated in vitro using ARPE-19 cells after 1 month under strict differentiation conditions; some processes, such as melanin accumulation as well as basal and maximal respiration, may require longer periods of maturation.
• Low-passage ARPE-19 kept in physiologically relevant-inducing culture conditions develop RPE-like phenotypic traits in a temporally regulated manner, with many core traits acquired relatively early during long-term culture.
• The metabolic and mitochondrial morphologic features of differentiated ARPE-19 cells closely mirror those found in native RPE, with increased reliance on oxidative phosphorylation, an increased mitochondrial mass, and increased propensity towards mitochondrial branching.

**What are the implications of the main findings?**

• Use of differentiated ARPE-19 cells as a viable model to study key RPE physiological functions should be accompanied by evidence of rigorous phenotypic and metabolic characterisation.
• Good practice and appropriately used differentiation protocols can ensure increased reliability and reproducibility for in vitro modelling employing ARPE-19 cells.

**Abstract:**

Background. ARPE-19 cells are widely used to investigate and model retinal pigmented epithelium (RPE). Naïve ARPE-19 cells do not fully replicate RPE features. Previous reports show that under defined conditions, ARPE-19 differentiate towards an RPE phenotype, with protocols relying on prolonged or shorter-term cultures, the latter of which do not achieve full pigmentation. In particular, bioenergetics and mitochondrial dynamics have been assessed in a limited manner to date. The present work aims to characterise ARPE-19 under long-term physiologically relevant phenotype-inducing culture conditions, monitoring the timeline of RPE phenotype acquisition, with a focus on mitochondrial function and bioenergetics alongside structural and functional markers. Methods. Low passage ARPE-19 were cultured for 4 months using low-serum, pyruvate and nicotinamide-supplemented DMEM. Monthly assessments measured pigmentation, TER, gene/protein expression, mitochondrial parameters, and metabolism. Results. ARPE-19 acquired relevant features early (1 month), including polarisation, expression of visual cycle and mitochondrial dynamic drivers, as well as increases in mitochondrial potential, mass/area and branching. Melanin accumulation was increased beginning at 1 month. Bioenergetic assays revealed a shift towards increased reliance on oxidative phosphorylation compared to glycolysis. Conclusions. Under these conditions, ARPE-19 acquired specific RPE features supporting functional assays after 1 month. Time-dependent accumulation of RPE-like features suggests that, for certain applications, later differentiation times are suitable. Appropriately differentiated ARPE-19 are a viable model to study key RPE physiological functions when accompanied by evidence of specific phenotypic and metabolic characterization.

## 1. Introduction

The retinal pigmented epithelium (RPE) is a cellular, highly polarised monolayer situated between the photoreceptors and choroid, establishing the outer layer of the human retina [1,2]. It is a key component of the retina, ensuring maintenance and functional crosstalk with its adjacent layers, the choriocapillaris and neuroretina. The RPE is essential for the survival and function of photoreceptor cells, and its dysfunction is implicated in a multitude of eye-related pathologies, including age-related macular degeneration (AMD) and inherited retinal diseases, for which there are currently no curative interventions [3]. Development of physiologically relevant in vitro cell-based models of RPE is crucial for supporting appropriate experiments aimed at understanding its biology in health and disease, as well as assessing potential treatments.

There are a number of currently available in vitro models for the study of RPE, including RPE cell lines, primary animal- and human-derived cell lines, iPSC-derived RPE models and organ-on-a-chip systems [4]. Among these, the cell line ARPE-19, derived from a primary human culture [5], has proven to be the most tractable, versatile, and enduring [4], as attested by its current 3118 entries in PubMed (retrieved October 2026). However, a large number of studies have inappropriately made use of naïve ARPE-19 cells, which do not fully express RPE functional markers, especially at low confluence or after repeated passages [6,7,8,9,10,11]. Conversely, it has been shown that, under appropriate culture conditions, ARPE-19 cells differentiate and acquire phenotypic features closely resembling native RPE cells [6,7,9,12,13,14,15,16].

Each ARPE-19 differentiation model study has assessed various phenotypic or metabolic features of RPE, in turn, showing various advantages and disadvantages, ranging from necessitating long-term culture up to 3 [7] or even 4 months [9], or in case of short-term (up to 1 month) protocols [13,14,16], evidencing that melanin pigmentation could not be fully replicated despite achieving polarity. Despite this fact, short-term differentiation protocols are useful tools for some RPE processes that do not depend strictly on pigmentation, or where pigment accumulation may be a hindrance, i.e., some imaging studies [14], as well as for assessing and screening various translational approaches, including payload delivery for gene therapies [15].

Commonalities among the protocols deployed for these models include the use of serum withdrawal, which is crucial to inducing the relevant transcriptome [6,9], as well as using extracellular matrix (ECM) coating for tissue culture surfaces, the latter being conducted by the majority of studies [7,9,12,13,14,15,16]. Various models leveraged different strategies in order to achieve a differentiated phenotype; these included using low passage ARPE-19 cell aliquots, which retain more features of native RPE compared to higher passages [9,12], and supplementing culture media with nicotinamide in order to boost mitochondrial development and metabolic activity [14,15,16].

Among the most important functions of the RPE is its metabolic role, being widely considered one of the most metabolically active tissues in the body [17]. Photoreceptors derive their energy by converting glucose to lactate through aerobic glycolysis in a Warburg effect-like process, relying on the RPE both for providing them with glucose from the choriocapillaris, as well as for uptaking and metabolising the resulting lactic acid [18,19,20,21]. To support this, RPE cells are endowed with an abundant mitochondriome [17] and conduct high levels of oxidative phosphorylation [22]. It is important, however, to note that all data pertaining to Warburg-like metabolism in the retina, the asymmetric metabolism of the RPE and neuroretina/outer photoreceptor segments (OS), as well as the central role of lactate in retinal metabolism, are derived from animal models, notably mice [18] and zebrafish [19]. Human models have relied on in vitro cell cultures and, as such, are marked by technical difficulties such as setting up co-cultures for the full replication of the biochemical niche established between the RPE and the OS.

While some of the in vitro ARPE-19-based RPE models discussed above have characterised various aspects of the metabolism and mitochondrial function, they have done so in a fairly limited fashion [14,16]. Of specific note, neither mitochondrial dynamics nor bioenergetics were ascertained comparatively, on a monthly basis, in a long-term follow-up of differentiated ARPE-19 grown under physiological conditions. Given the overall importance of these processes to RPE functionality in the broader context of its physiological role, these open questions need to be fully addressed. The importance of assessing the acquisition of a native RPE-like metabolic profile is further underscored by the fact that ARPE-19 cells have been shown to be useful for studying processes such as ER stress, oxidative stress, intracellular trafficking and secretion, as well as modelling and assessing further AMD-related changes in vitro [23,24,25,26,27,28].

The present work aims to build upon previously proposed models of long- and short-term ARPE-19 differentiation and conduct a thorough follow-up of up to 4 months in culture, assessing the timing and succession of the acquisition and maximal values of RPE features in vitro, aiming to characterise canonical features such as polarity, pigmentation, functional markers of adhesion, cellular transport, and visual cycle, while monitoring mitochondrial dynamics and bioenergetics. The findings provide a strong evidence basis for proposing an optimal threshold for the use of differentiated ARPE-19 cells in various functional assays, aimed at ascertaining relevant molecular, structural, and functional changes.

## 2. Materials and Methods

### 2.1. Cell Culture

Low passage (11–15) ARPE-19 cells (CRL-2302, ATCC, Manassas, VA, USA) were a gift from T. Michael Redmond’s lab at the NEI. Cell cultures were propagated and biobanked using standard proliferation medium [7,29], consisting of high-glucose DMEM without sodium pyruvate (Thermo Fisher, Waltham, MA, USA) supplemented with 10% FBS. For the experiments, ARPE-19 cells at passages 13–15 were seeded at confluence (1.3 × 10^5^/cm^2^) on MaxGel ECM (Merck, Darmstadt, Germany) human basement membrane-coated inserts and wells and, subsequently, grown in high-glucose DMEM supplemented with FBS 1%, sodium pyruvate 1 mM, nicotinamide 10 mM, HEPES 55 mM, penicillin 100 U/mL, streptomycin 100 µg/mL, and amphotericin 0.25 µg/mL for up to 4 months. Negative controls were grown using standard proliferation medium, as described above.

### 2.2. Measurement of Cell Pigmentation

Changes in cell morphology and pigmentation were monitored using brightfield microscopy on a DMi8 inverted microscope (Leica Microsystems, Wetzlar, Germany). Imaging analysis and quantification were performed using Fiji software (ImageJ 1.54p, Waye Rasband and contributors, National Institutes of Health, USA). Quantitative assessments of pigmentation were conducted by calculating mean grey values (MGV), subsequently normalised to surface values as integrated density (ID). These parameters inversely correlate with the black pixel values corresponding to melanin accumulation, i.e., lower MGV/ID values correspond to darker shades.

### 2.3. Measurement of Transepithelial Resistance (TER)

TER measurements were performed monthly across monolayers grown in ECM-coated transwells using the Milicell ERS-2 voltohmmeter (Merck) STX04 electrode within 10 min of removing the plate from the incubator. Blank measurements were performed using a well containing culture medium.

### 2.4. Gene Expression Assays

Relative gene expression was determined using qRT-PCR. RNA isolation was performed monthly for cells up to 4 months in culture using the RNeasy Plus Mini kits (Qiagen, Hilden, Germany). cDNA reverse transcription was performed using High Capacity RNA-to-cDNA kits (Thermo Fisher). qPCR was performed using PowerTrack SYBR Green Master Mix (Thermo Fisher). Primers for the following genes were used: *TYR*, *OCLN*, *BEST1*, *TJP1*, *RPE65* (for exons 2, 9–11, 10–11), *RBLP1*, *RDH5*, *MFN1*, *MFN2*, *OPA1*, *DRP1*, *FIS1*, and *GAPDH* was used as a housekeeping gene. All primers are available in the Supplementary Data Section (Supplementary Table S1) and were purchased from IDT (Coralville, IA, USA) and Merck. Acquisition was performed on a QuantStudio5 system (Thermo Fisher). Data were analysed using the 2^−ΔΔCt^ method.

### 2.5. Protein Expression Assays

Cells were harvested monthly using RIPA buffer supplemented with protease inhibitor cocktail (Calbiochem, San Diego, CA, USA) after prior resuspension, counting, and washing. Protein contents were measured using the Pierce Dilution-Free Rapid Gold BCA Protein Assay kit (Thermo Fisher), with 10 µg of protein sample loaded in each well, alongside a molecular weight marker, to perform SDS-PAGE (BioRad, Hercules, CA, USA), followed by immunoblotting. Nitrocellulose membranes were blocked for 2 h using Pierce Clear Milk Blocking Buffer (Thermo Fisher) diluted at 10% in distilled water, followed by probing with primary antibodies in Blocker BSA in TBS Concentrate diluted at 10% in TBS-T 0.05% buffer: TYR (1:1000, Santa Cruz Biotechnology, Dallas, TX, USA, sc-20035), OCLN (1:5000, Proteintech, Rosemont, IL, USA, 13409-1-AP), BEST1 (1:1000, Bio-Techne, Minnesota, MN, USA, NB300-164), ZO-1 (1:1000, Proteintech, 21773-1-AP), RPE65 (1:1000, Abcam, Cambridge, UK, ab13826), RDH5 (1:1000, Abcam, ab101457), CRALBP (1:1000, Abcam, ab15051), MFN1 (1:2000, Proteintech, 13798-1-AP), MFN2 (1:1000, Cell Signaling Technology, Danvers, MA, USA 9482), OPA1 (1:1000, Cell Signaling Technology, 80471), DRP1 (1:2000, Proteintech, 12957-1-AP), FIS1 (1:1000, Proteintech, 10956-1-AP), and α-tubulin (1:1000, Cell Signaling Technology, 3873) as a housekeeping protein. Detection was performed using horseradish peroxidase-conjugated secondary antibodies (1:2000) by chemiluminescence, using Clarity Western ECL Substrate (BioRad) and visualized using the ChemiDoc Touch Imaging System (BioRad). Densitometry values were assessed using the ImageLab software v6.1 (BioRad), with protein values in each sample normalised to α-tubulin and further against normalised controls to compute relative fold increase/decrease. All immunoblotting data are representative of 3 independent experiments.

### 2.6. Fluorescent Probe Assays for Mitochondrial Potential, Mass, and Oxidative Stress

Cells were seeded onto MaxGel ECM-coated black optical bottom Nunc 96-well microplates (Thermo Fisher) and maintained in culture for a duration of 4 months in differentiation medium formulated as above, i.e., low serum-containing, nicotinamide and pyruvate-supplemented DMEM. Short-term cultures were kept in proliferation medium formulated as above, i.e., pyruvate-free DMEM supplemented with 10% FBS, and served as experimental controls. Fluorescent probe-based assays were used to ascertain mitochondrial potential, mass, intracellular reactive oxygen species (ROS) production, mitochondrial ROS production, and superoxide ion production. Monthly, the cells were probed using the following fluorescent assays: TMRE-Mitochondrial Membrane Probe Assay (Abcam), Mitotracker Red FM (Thermo Fisher), ROS Detection Cell-Based Assay Kit (Cayman Chemical, Ann Arbor, MI, USA), CellROX Green (Thermo Fisher), and MitoSOX Green (Thermo Fisher), respectively, according to manufacturers’ instructions. The readouts were performed using a FLUOstar Omega microplate reader (BMG Labtech, Ortenberg, Germany). Following data acquisition, relative fluorescence intensity values (RFU) were normalised to cell counts.

### 2.7. Live Cell Microscopy Assays

Cells were seeded onto MaxGel ECM-coated 8-chamber glass bottom µ-slides (ibidi, Bayern, Germany) and kept as previously described, with controls grown in proliferation, 10% FBS-supplemented DMEM and differentiated cells in low serum-containing, nicotinamide and pyruvate-supplemented DMEM. Monthly, the cells were probed using MitoTracker Red FM fluorescent probes (Thermo Fisher) and observed on a Zeiss Celldiscoverer 7 (Carl Zeiss AG, Oberkochen, Germany) using widefield deconvolution mode. Imaging analysis and quantification were performed with Fiji software using the Mitochondria Analyzer plugin [30], assessing total and mean mitochondrial area, number of mitochondrial branches per field and per mitochondria, respectively, and branch length per mitochondria.

### 2.8. Assessment of Mitochondrial Cellular Bioenergetics

Bioenergetic parameters were assayed by measuring oxygen consumption rate (OCR) and extracellular acidification rate (ECAR) using a Seahorse XFe96 Analyzer (Agilent Technologies, Santa Klara, CA, USA). Cells were seeded at the same density as for previous experiments, 1.3 × 10^5^/cm^2^, onto MaxGel ECM-coated XF96 plates (Agilent Technologies), switched to differentiation medium, i.e., low serum-containing, nicotinamide- and pyruvate-supplemented DMEM, after attachment at 24 h and subsequently kept in culture for 3 months. Corresponding controls were seeded at the same time, switched to proliferation medium, i.e., pyruvate-free DMEM supplemented with 10% FBS, at 24 h, and subsequently kept in culture for the same duration. Assays were conducted at 1 day following the first medium change and, subsequently, on a monthly basis up to 3 months in culture. The Seahorse XF Real-Time ATP Rate and Mito Stress test kits (Agilent Technologies) were used at the designated assay time points, according to the manufacturer’s instructions. For the Mito Stress test, OCR was measured under basal conditions, followed by the sequential addition of mitochondrial stressors oligomycin (in order to measure mitochondrial ATP production), FCCP (mitochondrial uncoupler allowing for the measurement of maximal respiration), and, lastly, rotenone and actinomycin A (shutting down aerobic respiration and allowing glycolytic activity assessment). For the ATP Rate test, only injections of oligomycin and rotenone/actinomycin A were performed, in order to allow for ECAR measurements in the absence of FCCP. Measurements were subsequently normalised to cell counts.

### 2.9. Data and Statistical Analysis

Data analysis was conducted using GraphPad Prism 11.0.2 (GraphPad Software, San Diego, CA, USA) and Microsoft Excel. Values were expressed either as mean ± standard error of the mean (SEM) or mean ± standard deviation (SD), as specified. All results shown were obtained using three or more distinct biological replicates, as specified. Statistical tests were performed in accordance with the experimental design and distribution of each dataset, as appropriate: ANOVA with post hoc Tukey test for datasets meeting assumptions of parametric analysis, Kruskal–Wallis test for non-normally distributed datasets, the Jonckheere–Terpstra test and Spearman’s rho correlation for ordered differentiation data. All statistical tests were conducted using SPSS Statistics Version 31.0.2.0 software (IBM Corp., Armonk, NY, USA). Imaging data were subjected to quantitative analysis using Fiji and appropriate plugins and parameters, as specified. *p*-values of less than 0.05 were considered statistically significant. Additional thresholds for statistical significance were designated at 0.01 and 0.001. Diagrams were constructed in Canva, using visual elements from NIH BioArt Source and BioRender.

## 3. Results

### 3.1. Timeline for Attaining Pigmentation and RPE Morphology In Vitro by Low-Passage ARPE-19 Cells Grown Under Enhanced Physiological Conditions

**Figure 1:**
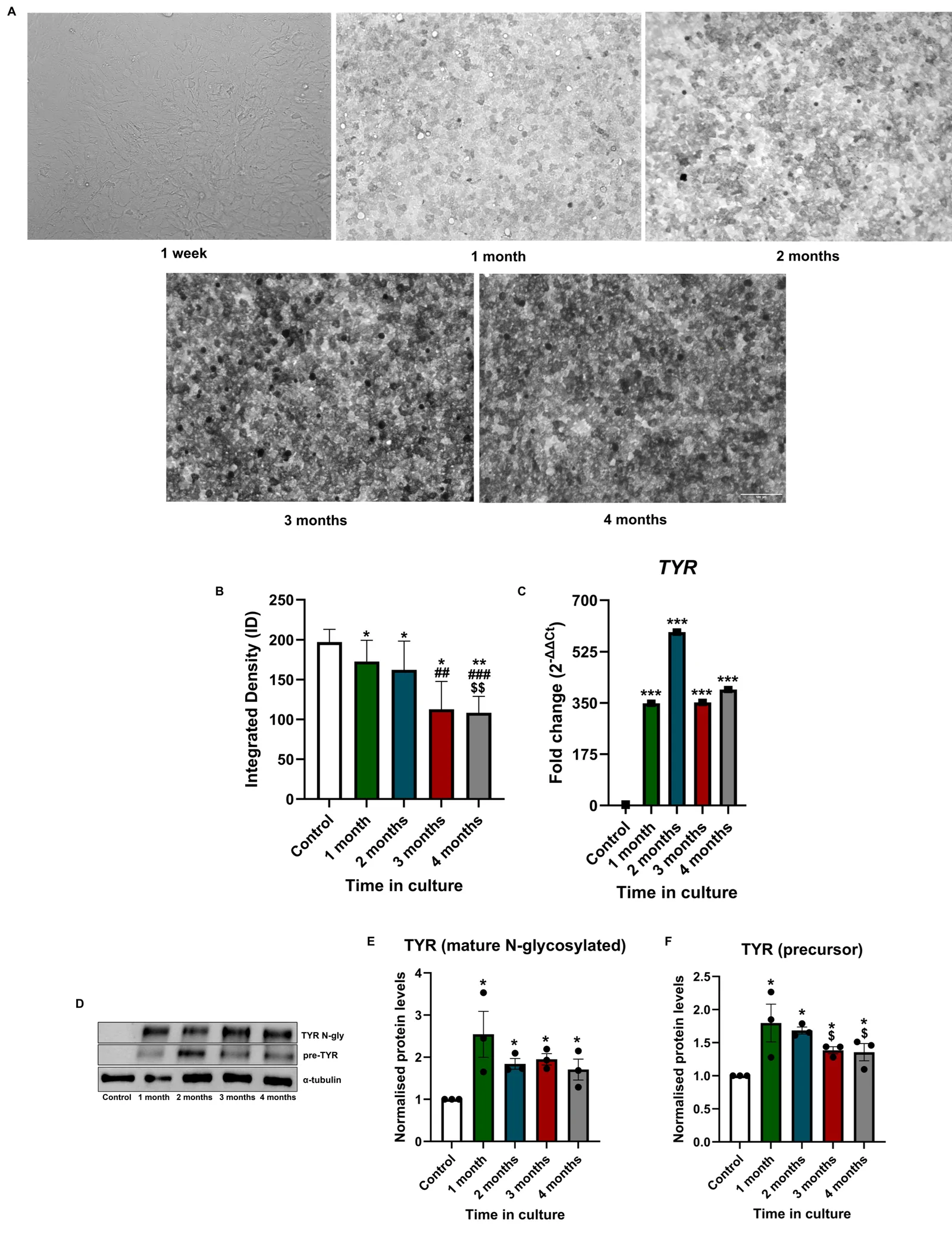
Characterisation of RPE morphology and melanin accumulation during long-term culture differentiation in physiological conditions. (**A**) Brightfield imaging accumulation of melanin in a time-dependent manner. Scale bar = 100 μm. (**B**) Integrated density (ID) values show a gradual time-dependent decrease (Spearman’s rho correlation, r_s_ = −0.695, *p* < 0.001), reaching their lowest points and plateauing at 3–4 months in culture. Data shown as mean values ± SEM (n ≥ 3). (**C**) Gene expression levels of *TYR* increased significantly at all surveyed time points compared to controls. Data shown as mean values ± SD (n ≥ 5). (**D**) Representative immunoblotting images of N-glycosylated and precursor TYR alongside housekeeping protein α-tubulin. (**E**) Protein expression levels of mature N-glycosylated TYR reveal significantly increased levels at all time points in culture. Data are shown as mean values ± SEM (n = 3). (**F**) Protein expression levels of precursor non-glycosylated TYR reveal significantly increased levels at all time points in culture. Data shown as mean values ± SEM (n = 3). ID data were analysed using Spearman’s rho correlation and the Jonckheere–Terpstra test; qRT-PCR data were analysed using ANOVA with post hoc Tukey test and immunoblotting data were analysed using the Jonckheere–Terpstra test. * *p* < 0.05 vs. control, ** *p* < 0.01 vs. control, *** *p* < 0.001 vs. control; ## *p* < 0.01 vs. 1 month, ### *p* < 0.001 vs. 1 month; $ *p* < 0.05 vs. 2 months, $$ *p* < 0.01 vs. 2 months.

Low-passage ARPE-19 cells grown under physiological-inducing conditions acquire RPE pigmentation and morphology in vitro. The cells started acquiring specific RPE polyhedral, cobblestone-like morphology, as well as melanin pigmentation, as early as one month in culture, increasing at two months, and plateauing at 3 months in culture (Figure 1A). ID was computed from MGV normalised to surface, therefore inversely correlated with the black values corresponding to melanin accumulation (Figure 1B). ID values were found to decrease significantly across differentiation time, consistent with a time-dependent increase in pigment with maturation (Spearman’s rank correlation, r_s_ = −0.695, *p* < 0.001). ID values were found to be significantly lower compared to controls at all time points ascertained, progressively and significantly higher at 3 and 4 months compared to 1 and 2 months in culture, where they reached a plateau. This suggests a pattern of incremental, time-dependent accumulation of melanin up until 3 months in culture when cells are grown under physiological conditions.

Tyrosinase, the rate-limiting enzyme of melanin synthesis [31,32], was characterised in regard to gene and protein expression levels. The *TYR* gene was overexpressed at all time points compared to controls (Figure 1C). TYR protein expression levels were determined for both its N-glycosylated (Figure 1E) as well as its non-glycosylated precursor (Figure 1F) forms. The glycosylated form of tyrosinase is catalytically active and has been shown to be directly correlated with melanin pigmentation levels [33,34], while the non-glycosylated or less-glycosylated form is usually considered its immature precursor molecule. Consequently, N-glycosylated TYR was found to be elevated at all time points surveyed (Figure 1E), while precursor TYR was found to be upregulated at all time points in culture, peaking at 1–2 months (Figure 1F), indicative of sustained production preceding and underpinning the gradual melanosome accumulation.

### 3.2. RPE Polarisation Is Acquired Early in Long-Term Physiological Phenotype-Inducing Culture Conditions

**Figure 2:**
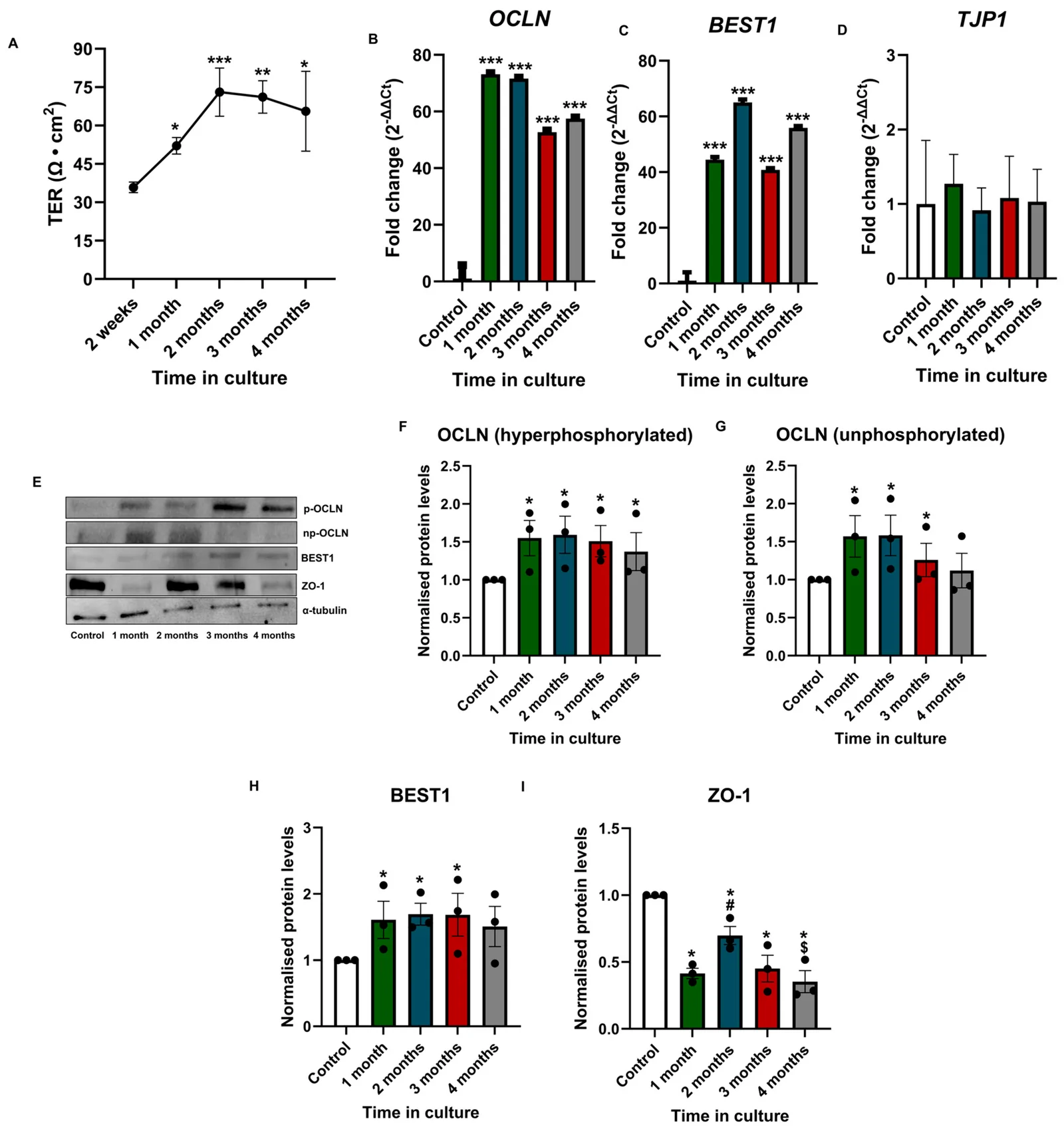
Polarisation of RPE during long-term culture in physiological conditions. (**A**) TER increased in long-term culture, achieving significantly elevated polarity levels at 1 month in culture. Data shown as mean values ± SEM (n ≥ 4). (**B**) Gene expression levels of *OCLN* are upregulated at all surveyed time points in culture. Data shown as mean values ± SD (n ≥ 5). (**C**) Gene expression levels of *BEST1* are significantly increased at all time points compared to controls. Data shown as mean values ± SD (n ≥ 5). (**D**) Gene expression levels of *TJP1* were not significantly modified compared to controls at all surveyed time points. Data shown as mean values ± SD (n ≥ 5). (**E**) Representative immunoblotting images of hyperphosphorylated and unphosphorylated/less phosphorylated OCLN, BEST1, and ZO-1 alongside housekeeping protein α-tubulin. (**F**) Protein expression levels of hyperphosphorylated OCLN show significantly increased levels at all time points in culture. Data shown as mean values ± SEM (n = 3). (**G**) Protein expression levels of unphosphorylated/less phosphorylated OCLN show significantly increased levels at 1–3 months. Data shown as mean values ± SEM (n = 3). (**H**) Protein expression levels of BEST1 show significantly increased levels at 1–3 months. Data shown as mean values ± SEM (n = 3). (**I**) Protein expression of ZO-1 has been documented at all surveyed time points, with differentiated cells showing significantly lower levels compared to controls. Data shown as mean values ± SEM (n = 3). TER data were analysed using the Kruskal–Wallis test, qRT-PCR data were analysed using ANOVA with post hoc Tukey test, and immunoblotting data were analysed using the Jonckheere–Terpstra test. * *p* < 0.05 vs. control, ** *p* < 0.01 vs. control, *** *p* < 0.001 vs. control; # *p* < 0.05 vs. 1 month; $ *p* < 0.05 vs. 2 months.

TER was monitored beginning at 2 weeks in culture and, subsequently, on a monthly basis. The cultured cells reached TER values suggestive of polarisation and remained significantly elevated throughout the follow-up (Figure 2A), exhibiting statistically significant increases compared to the 2-week mark at every assessed timepoint. While TER for primary RPE cells routinely surpassed values of 400 Ω·cm^2^ in culture [35,36,37,38], ARPE-19 cells thus far rarely achieved TER values greater than 40 Ω·cm^2^ [7,13,36,37,38], with Dunn et al. reporting a maximum of 100 Ω·cm^2^ at 4–6 weeks in culture following their initial isolation [5]. The current findings are, to our knowledge, among the highest documented values for ARPE-19 cells in culture [37].

In order to study molecular determinants of RPE polarity, we ascertained the gene and protein expression levels of occludin (OCLN), zonula occludens-1 (ZO-1), and bestrophin-1 (BEST1). Occludin is a critical component of the tight junction complexes in the RPE, regulating the barrier function and permeability of the monolayer [39] and, therefore, accounting for apical–basal asymmetry and the uneven distribution of ionic species across the tissue [39,40]. The choice of OCLN as a marker of tight junctions (TJs) was motivated by its specificity to these junctions [41], taking into account the integral role of TJs in the structure and function of the blood–retina barrier (BRB) [42], of which the RPE is an integral part. Occludin is found in two forms, phosphorylated/hyperphosphorylated and unphosphorylated/less phosphorylated, the former being the membrane-bound integral active form incorporated in the structure of TJs [43,44], while the latter remains soluble inside the cytosol, with dephosphorylation regulating paracellular permeability and suggesting tight junction remodelling or disruption [44,45]. ZO-1 is a cytoplasmic component of TJs and adherens junctions, serving as an adaptor and scaffold tethering transmembrane and cytoplasmic proteins [46,47]. While often used as a maturation marker of in vitro RPE monolayers [38], its localisation is not strictly specific to RPE TJs [46], and its disruption, while increasing BRB permeability to large solutes, does not abolish TER [47].

BEST1 is an anion-selective channel activated by intracellular Ca^2+^, highly expressed in the RPE, acting as Cl^−^ channels, as well as regulators of voltage-gated Ca^2+^ channels, and an important determinant of transepithelial fluid transport [48,49,50]. BEST1 is selectively localised on the basolateral membrane of the RPE [49].

OCLN and BEST1 gene expression levels were found to be significantly upregulated at all time points ascertained, beginning at 1 month (Figure 2B,C, respectively). Hyperphosphorylated OCLN protein expression mirrored this pattern closely (Figure 2F), exhibiting increased levels at all time points surveyed, and unphosphorylated OCLN protein expression was significantly upregulated at months 1–3 (Figure 2G). Given that TER levels peak and remain at sustained levels after 1 month in culture, this is suggestive of active TJ remodelling, with unphosphorylated/less phosphorylated OCLN increases balancing hyperphosphorylated OCLN levels during months 1–3. BEST1 protein was found to be upregulated at 1–3 months in culture (Figure 2H).

By contrast, *TJP1*, encoding ZO-1, was not significantly upregulated at any of the time points surveyed (Figure 2D), with ZO-1 protein detected at all time points surveyed, but downregulated compared to controls (Figure 2I). This is consistent with previous findings, which outlined that in primary cultures of chicken RPE/choroid, occludin expression remained stable or registered slight increases, while total ZO-1 immune reactivity decreased [51]. It has been posited that the mechanistic basis of this finding is the fact that ZO-1 is present in the initial stages of TJ assembly, but that throughout organogenetic development, total ZO-1 is decreased as TJs mature, due to shifts in expression of its splice variants [52], as documented via transcriptomic studies [53]. Moreover, in vitro studies of hfRPE long-term cultures have described a similar pattern, with ZO-1 peaking at 10 days in culture and declining thereafter with subsequent maturation [54]. In summation, the apical junctional complex undergoes complex remodelling during BRB formation, including a marked reduction in ZO-1 immune reactivity, parallel to paracellular permeability decrease, while *TJP1* abundance and junctional localisation are also not temporally consistent [51,52,53,54]. Its accurate dynamic study during RPE differentiation/maturation is additionally complicated by the presence of multiple temporally-conditioned splice forms [52], as well as its nuclear translocation and accumulation throughout various developmental stages [55].

Taken together, the data suggest that the achievement of sustained levels of RPE polarisation in culture and its stability throughout the follow-up was chiefly underpinned by an upregulation of functionally relevant elements reinforcing the barrier function of the mature RPE, such as OCLN, in conjunction with increases in the maturation of the transmembrane transporter apparatus [56]. The presence of ZO-1 was documented throughout the follow-up, but was much stronger at an early stage, potentially indicative of TJ/apical complex assembly.

### 3.3. Key Components of Visual Cycle Regulation Are Upregulated Early During Long-Term Culture in Physiological Conditions

Expression levels of RPE-specific visual cycle genes and proteins were assessed, notably RPE65, an isomerohydrolase and a crucial marker of RPE tissue involved in regenerating 11-cis retinol [57], alongside RDH5, its downstream effector that oxidises 11-cis retinol before its shuttling towards the photoreceptors [58], and CRALBP, which acts downstream of RDH5 [58], supporting 11-cis-retinaldehyde production in the RPE and its delivery to the photoreceptor cells. It is also notable that when RPE65 is subjected to oxidative stress or light exposure, it undergoes a process of cleavage, yielding the truncated fragment RPE45 [59], whose functional role and significance have not yet been fully specified. In vitro differentiated models of RPE tend to exhibit low levels of expression of visual cycle genes, notably RPE65 [16]. RPE65 is a large gene, containing 14 coding exons and presenting multiple normal and pathological transcriptional variants [16,60]. Because of this, a number of primer pairs identifying various regions of the RPE65 mRNA transcript were employed, targeting exons 2, 9–11 and 10–11, with the latter two spanning exon–exon junctions to avoid any potential gDNA amplification.

**Figure 3:**
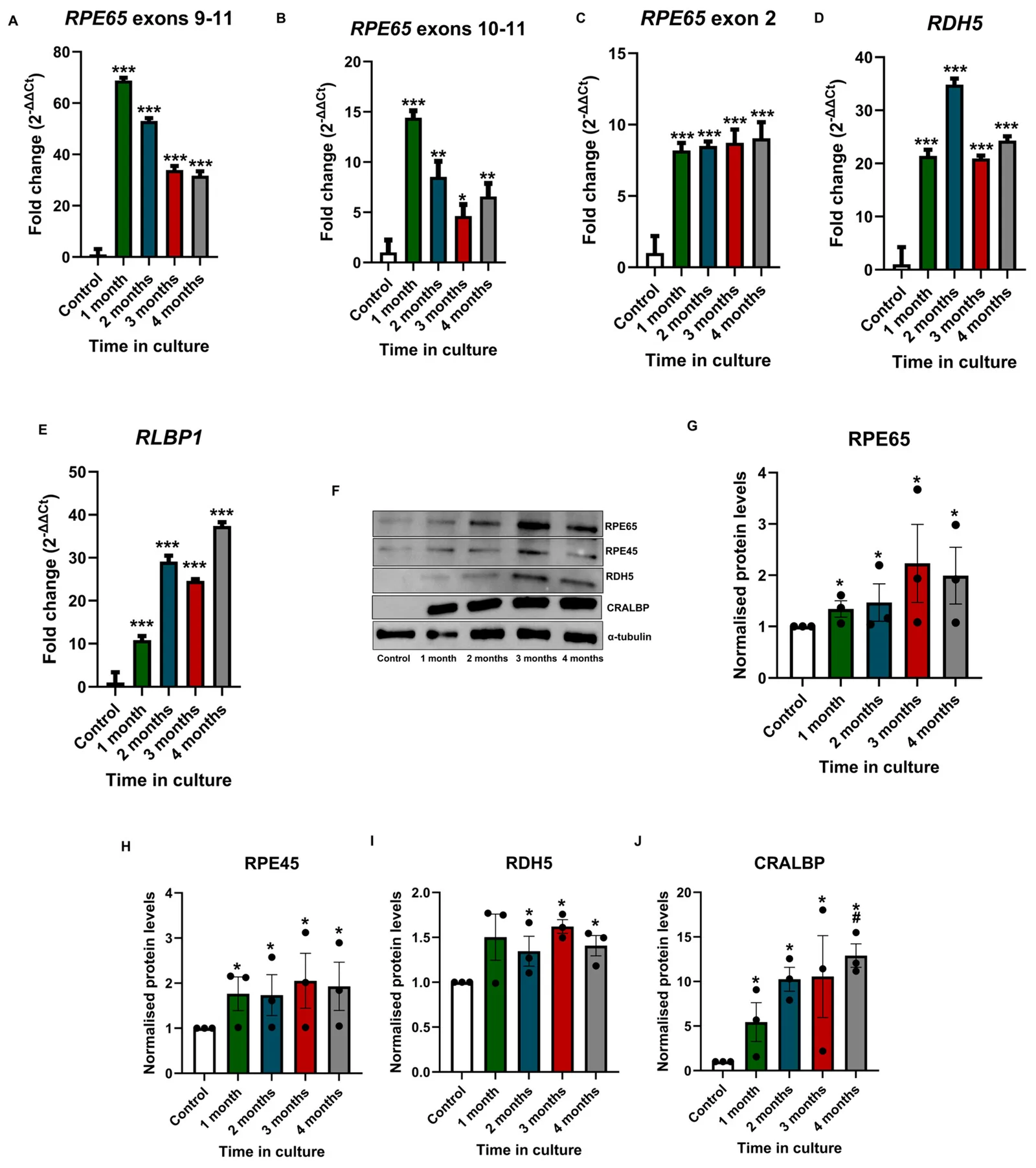
Assessment of gene and protein expression levels of visual cycle components. (**A**–**C**) Gene expression levels of *RPE65* exons 9–11, 10–11, and 2, respectively, show upregulation at all surveyed time points compared to controls. Data shown as mean values ± SD (n ≥ 5). (**D**) Gene expression levels of *RDH5* show significant overexpression at all surveyed time points. Data shown as mean values ± SD (n ≥ 5). (**E**) Gene expression levels of *RBLP1*, encoding CRALBP, show overexpression at all time points in culture compared to controls. Data shown as mean values ± SD (n ≥ 5). (**F**) Representative immunoblotting images of RPE65, RPE45, RDH5, and CRALBP alongside housekeeping protein α-tubulin. (**G**) Protein expression levels of RPE65 show significantly higher levels at all time points in culture compared to controls. Data shown as mean values ± SEM (n = 3). (**H**) Protein expression levels of RPE45 show significantly higher levels at all time points in culture compared to controls. Data shown as mean values ± SEM (n = 3). (**I**) Protein expression levels of RDH5 show significantly higher levels after 2 months. Data shown as mean values ± SEM (n = 3). (**J**) Protein expression levels of CRALBP show significantly higher expression at all time points in culture compared to controls. qRT-PCR data were analysed using ANOVA with post hoc Tukey test, and immunoblotting data were analysed using the Jonckheere–Terpstra test. * *p* < 0.05 vs. control, ** *p* < 0.01 vs. control, *** *p* < 0.001 vs. control; # *p* < 0.05 vs. 1 month.

Increased RPE65 gene expression was found at all ascertained time points for exons 9–11 (Figure 3A), 10–11 (Figure 3B), and 2 (Figure 3C). RPE65 protein expression levels were found to be significantly increased at all time points in long-term culture compared to controls (Figure 3G), while RPE45 was increased for all experimental groups (Figure 3H), suggesting that intermittent exposure to light during culture and harvesting may lead to an underestimation of actual RPE65 cellular levels. RDH5 gene expression levels were found to be upregulated at all time points in culture (Figure 3D), with protein levels significantly increased beginning at 2 months (Figure 3I). CRALBP gene (Figure 3E) and protein (Figure 3J) expression levels were consistently increased beginning at 1 month in culture.

### 3.4. Regulators of Both Mitochondrial Fusion and Fission Increase with RPE Differentiation, Suggestive of Active Mitochondrial Remodelling

Regulators of mitochondrial dynamics, comprising mitochondrial fusion and fission-related factors, were assessed at gene and protein expression levels. Mitochondrial fusion is regulated by GTPases known as mitofusins (MFN1 and MFN2), which are essential to coordinating fusion of the outer mitochondrial membrane (OMM), and the optic atrophy 1 protein (OPA1), which regulates the fusion of the inner mitochondrial membranes (IMM) [61,62]. OPA1 is a crucial regulator of mitochondrial morphology, which is present as 8 isoforms in humans, which are further grouped into long, membrane-anchored forms (L-OPA1), playing the canonical pro-fusion role, and short soluble forms (S-OPA1), which have been hypothesised to be a trigger of fission [63,64]. MFN2 and OPA1, in addition to regulating mitochondrial fusion and integrity, also play roles in bioenergetics, intracellular signalling, and mitophagy [65]. Mitochondrial fission is regulated by the DRP1/FIS1 interaction, whereby the activated GTPase DRP1 translocates to the OMM, interacting with the binding protein FIS1 [66].

Fusion and fission occur in a simultaneous and continuous fashion, with balance between the two accounting for overall mitochondrial morphology and ensuring the replacement of damaged or lost components, both processes being essential to survival and development [63,66]. Excessive fission is a feature of neurodegenerative conditions such as Alzheimer’s disease [67].

**Figure 4:**
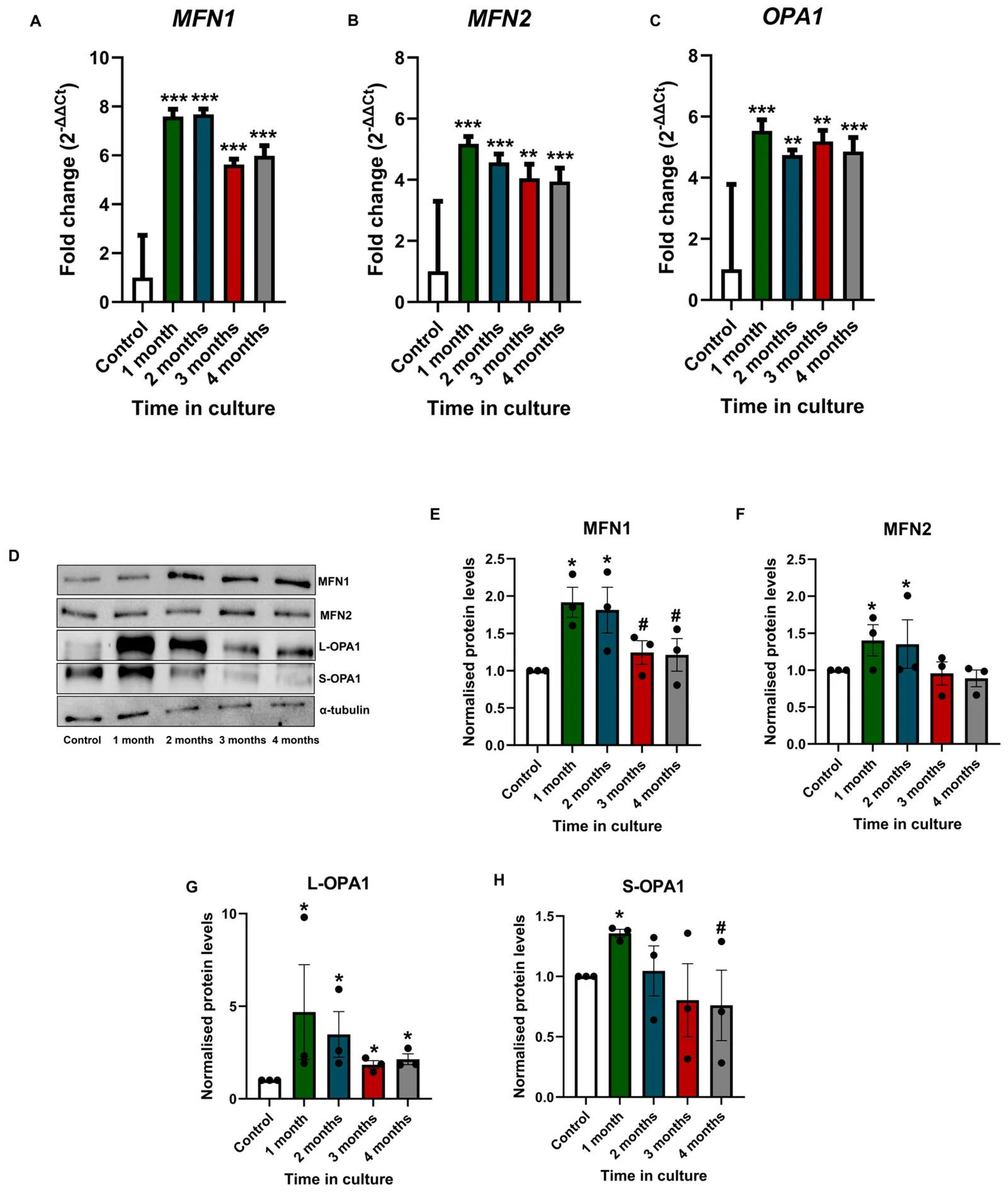
Assessment of gene and protein expression levels of mitochondrial fusion regulators. (**A**) Gene expression levels of *MFN1* show upregulation compared to controls at all time points surveyed. Data shown as mean values ± SD (n ≥ 5). (**B**) Gene expression levels of *MFN2* show upregulation compared to controls at all surveyed time points. Data shown as mean values ± SD (n ≥ 5). (**C**) Gene expression levels of *OPA1* show upregulation compared to controls at all surveyed time points. Data shown as mean values ± SD (n ≥ 3). (**D**) Representative immunoblotting images of MFN1, MFN2, L-OPA1, and S-OPA1 alongside housekeeping protein α-tubulin. (**E**) Protein expression levels of MFN1 show significantly increased levels at months 1–2 compared to controls, with protein levels significantly lower compared to month 1 at 3 and 4 months. Data shown as mean values ± SEM (n = 3). (**F**) Protein expression levels of MFN2 are significantly increased during the first two months in culture compared to controls. Data shown as mean values ± SEM (n = 3). (**G**) Protein expression levels of L-OPA1 are increased at all time points in culture compared to controls. Data shown as mean values ± SEM (n = 3). (**H**) Protein expression levels of S-OPA1 increased at 1 month compared to controls. Data shown as mean values ± SEM (n = 3). qRT-PCR data were analysed using ANOVA with post hoc Tukey test, and immunoblotting data were analysed using the Jonckheere–Terpstra test. * *p* < 0.05 vs. control, ** *p* < 0.01 vs. control, *** *p* < 0.001 vs. control; # *p* < 0.05 vs. 1 month.

**Figure 5:**
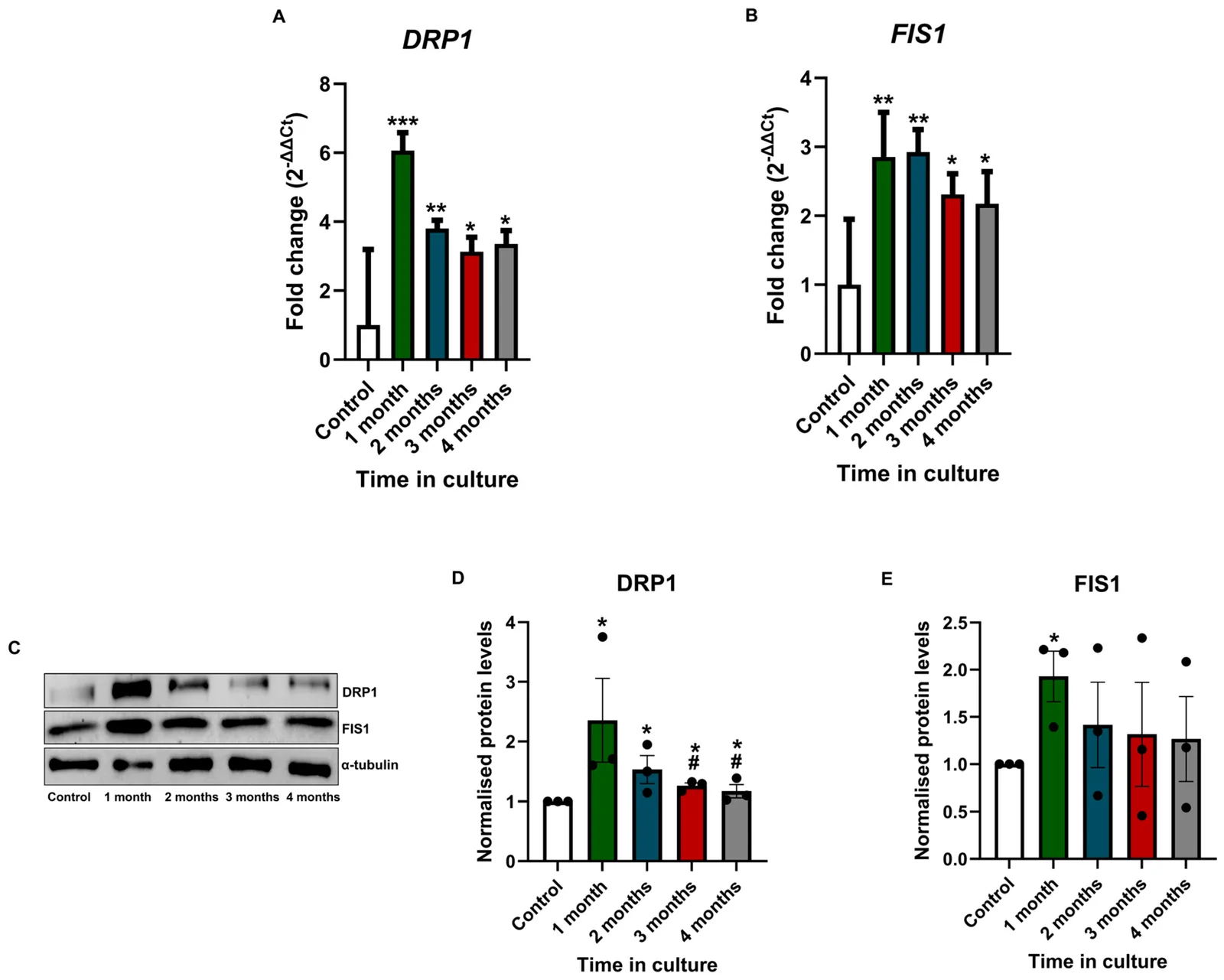
Assessment of gene and protein expression levels of mitochondrial fission regulators. (**A**) Gene expression levels of *DRP1* show upregulation compared to controls at all surveyed time points. Data shown as mean values ± SD (n ≥ 5). (**B**) Gene expression levels of *FIS1* show upregulation compared to controls at all surveyed time points. Data shown as mean values ± SD (n ≥ 3). (**C**) Representative immunoblotting images of DRP1 and FIS1 alongside housekeeping protein α-tubulin. (**D**) Protein expression levels of DRP1 are significantly increased at all surveyed time points compared to controls, with values at months 3–4 decreased compared to month 1. Data shown as mean values ± SEM (n = 3). (**E**) Protein expression levels of FIS1 are increased at 1 month in culture compared to controls. Data shown as mean values ± SEM (n = 3). qRT-PCR data were analysed using ANOVA with post hoc Tukey test, and immunoblotting data were analysed using the Jonckheere–Terpstra test. * *p* < 0.05 vs. control, ** *p* < 0.01 vs. control, *** *p* < 0.001 vs. control; # *p* < 0.05 vs. 1 month.

Gene expression levels of the mitochondrial fusion factors *MFN1*, *MFN2,* and *OPA1* exhibit significant upregulation at all assayed time points (Figure 4A–C). Protein expression levels showed an increase in MFN1 (Figure 4E) and MFN2 (Figure 4F) at 1–2 months in culture. Both L-OPA1 and S-OPA1 forms were detected, with the former increased at all time points in culture (Figure 4G) and the latter at 1 month (Figure 4H). Mitochondrial fission factors *DRP1* and *FIS1* gene expression levels were found to be overexpressed at all assayed time points (Figure 5A,B), with DRP1 protein levels significantly increased at all surveyed time points (Figure 5D), and FIS1 protein levels significantly increased at 1 month (Figure 5E).

Taken together, the data are suggestive of a high degree of temporally-conditioned mitochondrial remodelling activity occurring throughout the entire span of the follow-up, but notably peaking at months 1–2 in culture, suggestive of a strong early remodelling phase of increased fusion/network construction, as well as a close balance and crosstalk between mitochondrial fusion and fission throughout the follow-up. Notably, persistent L-OPA is suggestive of maintenance of structural IMM architecture and cristae integrity [68], even after OMM expansion concludes during early differentiation.

### 3.5. Mitochondrial Mass and Potential Increase During RPE Differentiation, Accompanied by a Steady Increase in ROS Generation

Mitochondrial membrane potential and changes in total mitochondrial mass were measured using fluorescent probe-based assays TMRE (Abcam) and MitoTracker Red FM (Thermo Fisher). TMRE is a lipophilic fluorescent dye that accumulates in active mitochondria as a consequence of the negative overall charge produced by mitochondrial potential and disperses when the potential is reduced or abolished. MitoTracker has similar properties, being positively charged and accumulating in mitochondria as a consequence of the presence of negative potential; the difference lies in its increased stability, as it forms covalent bonds with mitochondrial proteins, and therefore stains the mitochondria even after mitochondrial potential is abolished. Because of these differences, the former is used chiefly as a measure of mitochondrial potential in live cells, while the latter has the added advantage of being a more reliable indicator of mitochondrial mass.

**Figure 6:**
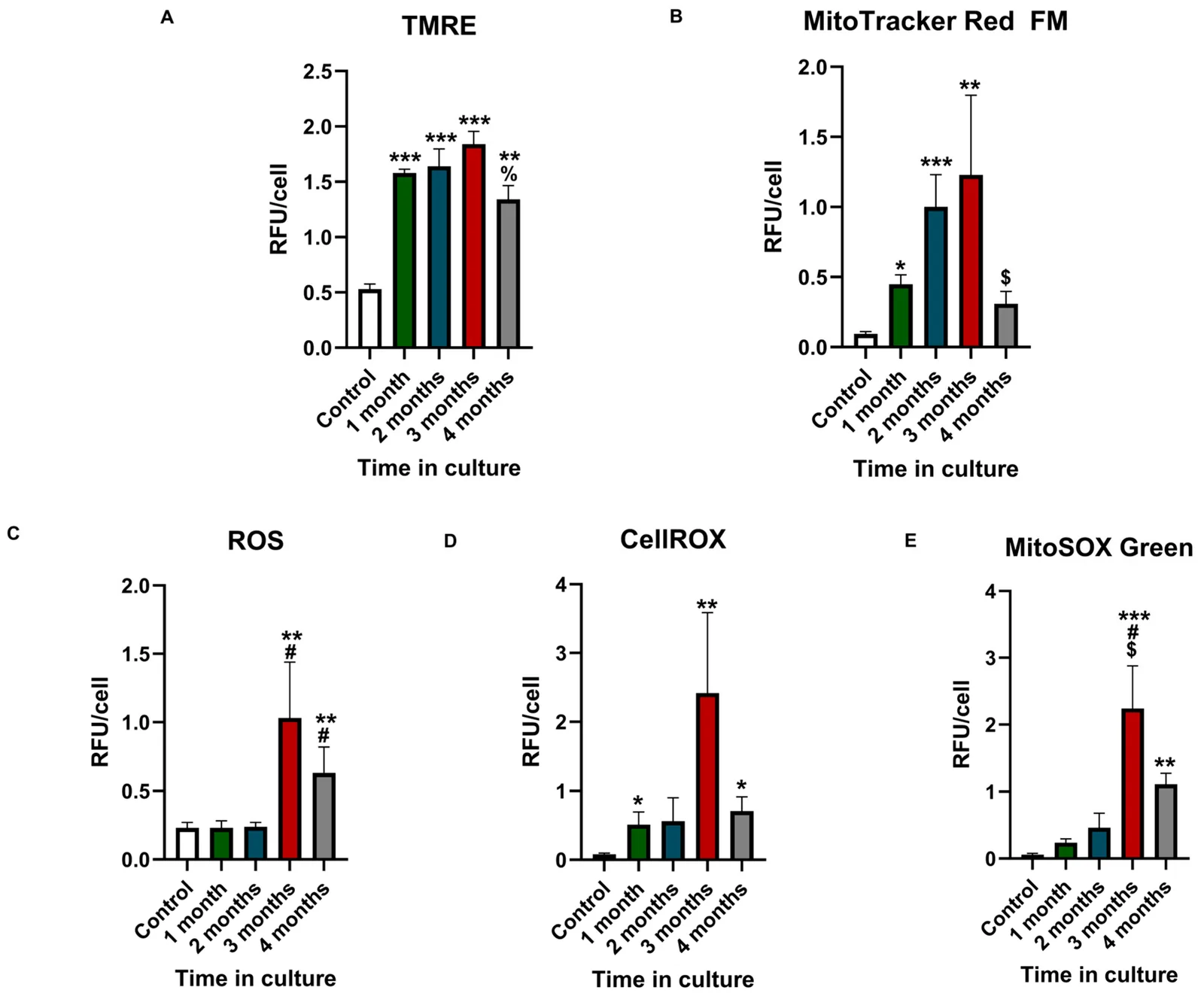
Mitochondrial mass, potential, and the production of reactive oxygen species. (**A**) TMRE assays normalised to cell counts show a relative increase in fluorescence intensity at all time points in culture for differentiated cells, followed by a relative decline at 4 months. (**B**) MitoTracker Red FM assays normalised to cell counts show a relative increase in fluorescence intensity at months 1–3 in culture in differentiated cells, followed by a decline at 4 months. (**C**) Mitochondrial ROS assays normalised to cell counts show increased fluorescence intensity at 3 and 4 months in culture compared to controls and 1 month. (**D**) Cytoplasmic ROS production measured using the CellROX assay normalised to cell counts revealed an increase in fluorescence intensity at 1, 3, and 4 months in culture. (**E**) Mitochondrial superoxide production measured using the MitoSOX assay normalised to cell counts revealed increased fluorescence intensity at 3 and 4 months in culture compared to controls. Data shown as mean values ± SEM (n ≥ 3). Normalised RFU values were analysed using the Kruskal–Wallis test. * *p* < 0.05 vs. control, ** *p* < 0.01 vs. control, *** *p* < 0.001 vs. control; # *p* < 0.05 vs. 1 month; $ *p* < 0.05 vs. 2 months; % *p* < 0.05 vs. 3 months.

Monthly assays conducted according to the manufacturer’s instructions, with RFU values normalised to cell counts subsequently revealed that mitochondrial potential measured using the TMRE assay is significantly increased at all time points in culture, followed by a relative decline at 4 months compared to month 3 in culture (Figure 6A). MitoTracker Red FM probes revealed a similar pattern, with mitochondrial mass significantly increased at months 1–3 in culture followed by a relative decline compared to month 2 (Figure 6B). Taken together, the data suggest that the differentiated RPE cells display the highest overall mitochondrial mass and potential relatively early in the follow-up, maintaining stable levels of increased mitochondrial mass and potential for at least 3 months in culture.

Reactive oxygen species (ROS) were monitored throughout the follow-up using three fluorescent-based assays, with the Cayman ROS assay being used to monitor mitochondrial matrix ROS production, CellROX (Thermo Fisher) to monitor overall ROS production in the cytoplasm, and MitoSOX to specifically measure mitochondrial production of superoxide.

Mitochondrial, cytoplasmic ROS, and mitochondrial production of superoxide normalised to cell counts followed overall similar generation patterns, peaking at 3 months in culture and remaining at elevated levels at 4 (Figure 6C–E), possibly as a result of increases in overall metabolic activity, especially oxidative phosphorylation.

### 3.6. Mitochondrial Area and Branching Are Subject to Major Shifts Throughout RPE Differentiation in Long-Term Culture

**Figure 7:**
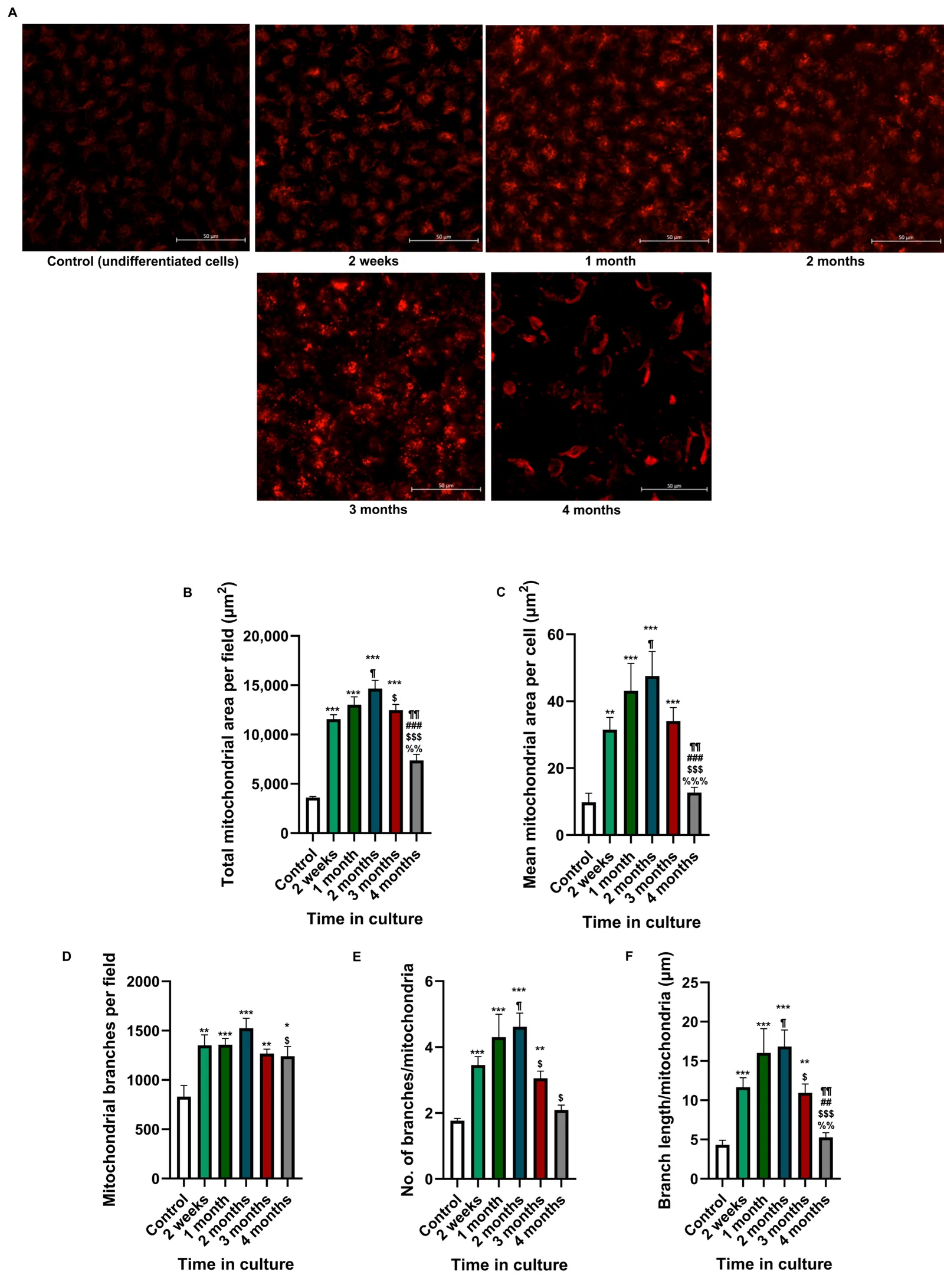
Measurement of mitochondrial area and branching. (**A**) Widefield deconvolution microscopy fluorescence imaging of MitoTracker Red FM-stained mitochondria in live RPE cells reveals substantial changes in mitochondrial area, distribution, and degree of branching in long-term differentiation-inducing culture. Scale bar = 50 μm. (**B**,**C**) Mitochondrial area assessed both as total area per field and mean area per cell, respectively, revealing statistically significant increases beginning at 2 weeks in culture, peaking, in both cases, at 2 months. Data shown as mean values ± SEM (n ≥ 5). (**D**) Mitochondrial branches per field are significantly increased at all time points surveyed for the differentiated cells. Data shown as mean values ± SEM (n ≥ 5). (**E**) The number of branches per mitochondria are significantly increased from 2 weeks until 3 months in culture. Data shown as mean values ± SEM (n ≥ 5). (**F**) Branch length per mitochondria was found to be significantly increased at 2 weeks–3 months in culture. Data shown as mean values ± SEM (n ≥ 5). Data were normalised per field, cell, or mitochondria, as indicated. Statistical analysis was conducted using the Kruskal–Wallis test. * *p* < 0.05 vs. control, ** *p* < 0.01 vs. control, *** *p* < 0.001 vs. control; ¶ *p* < 0.05 vs. 2 weeks, ¶¶ *p* < 0.01 vs. 2 weeks; ## *p* < 0.01 vs. 1 month, ### *p* < 0.001 vs. 1 month; $ *p* < 0.05 vs. 2 months, $$$ *p* < 0.001 vs. 2 months; %% *p* < 0.01 vs. 3 months, %%% *p* < 0.001 vs. 3 months.

Following plate-based assays revealing increases in mitochondrial mass, potential, and reactive oxygen species production as a consequence of RPE differentiation and immunoblotting data suggesting ongoing mitochondrial remodelling, live cell widefield deconvolution microscopy assays were further conducted to monitor dynamic changes in mitochondrial area, shape, and distribution. The imaging data revealed important shifts in the surface area, as well as the degree of mitochondrial branching, the end-product of mitochondrial fusion–fission interplay (Figure 7A). Mitochondrial area per field, as well as average mitochondrial area per cell, were found to increase in a time-dependent fashion starting as early as 2 weeks in culture, reaching a peak at 2 months and exhibiting a relative decrease at 3 and 4 months with regard to total area and at 4 months with regard to mean area per cell, approaching the values measured for controls with regard to mean area per cell (Figure 7B,C, respectively). These findings are consistent with measurements conducted using mitochondrial probes on plate-based assays (Figure 6A,B), as well as the pattern of fusion/fission protein marker expression, suggestive of an early intense remodelling phase (Figure 4E–H and Figure 5D,E).

Mitochondrial branching was found to significantly increase at 2 weeks, plateauing until 2 months, followed by a slight significant decrease at 4 months per field (Figure 7D). The number of branches per mitochondria increased significantly beginning as early as 2 weeks, peaking at 2 months in culture, followed by a relative decline at 3 and 4 months in culture (Figure 7E). Branch length per mitochondria started increasing at 2 weeks in culture, peaked at 2 months in culture, exhibited relative decline at 3 months in culture, remained significantly higher than controls, and finally declined to a level close to that in controls at 4 months in culture (Figure 7F). These findings provided insight into the pattern of mitochondrial area expansion highlighted in Figure 6A,B, as a product of mitochondrial fusion and ramification, and are further supported by the transient increase in OMM fusion markers documented during early stages of differentiation (Figure 4E,F).

Taken together, the data suggest that mitochondrial area and branching start increasing very early during follow-up, at 2 weeks, reaching peak levels at 2 months in culture. This is consistent with the pattern of gene and protein expression of mitochondrial fusion and fission markers, suggestive of an early network formation phase characterised by coordinated activation of both processes.

### 3.7. Differentiated RPE Cells Exhibit Increased Aerobic Cell Respiration and ATP Production

Having established that differentiation increases mitochondrial content, potential, dynamics and branching, we measured mitochondrial bioenergetics using the XF Real-Time ATP Rate and Mito Stress assays.

**Figure 8:**
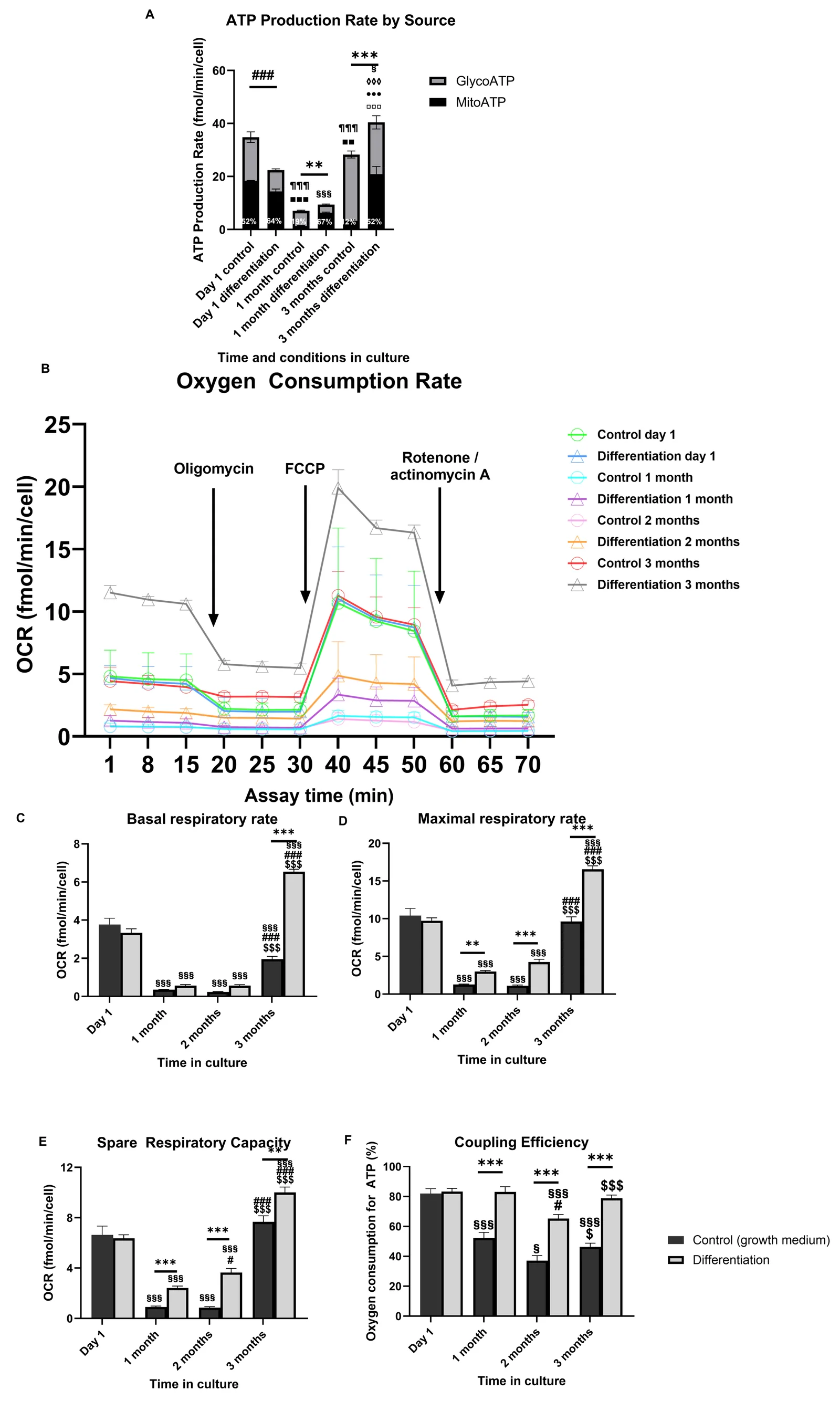
Bioenergetics of differentiated ARPE19 cells in long-term culture. (**A**) Comparing mean differences between mitoATP and glycoATP production at day 1, 1 month, and 3 months, for cells kept in growth medium (controls) and differentiation medium, reveals increased reliance on mitoATP production in differentiated cells. Data shown as mean ± SEM (n ≥ 3), ** *p* < 0.01, *** *p* < 0.001 mitoATP, ### *p* < 0.001 glycoATP, § *p* < 0.05 vs. day 1 differentiation mitoATP, §§§ *p* < 0.001 vs. day 1 differentiation mitoATP, ◊◊◊ *p* < 0.001 vs. 1 month mitoATP, ¶¶¶ *p* < 0.01 vs. day 1 control mitoATP, ••• *p* < 0.001 vs. day 1 differentiation glycoATP, ▫▫▫ *p* < 0.001 vs. 1 month glycoATP. (**B**) During the Seahorse MitoStress assay, mitochondrial stressors oligomycin, FCCP, rotenone, and antimycin A were sequentially injected while OCR was measured. (**C**) Bioenergetic assays evaluating basal and maximal respiration, spare respiratory capacity, and coupling efficiency were performed at day 1, then at 1, 2, and 3 months for ARPE-19 cells in growth medium (controls) and in differentiation-inducing medium. Differentiated cells showed significant increases in basal mitochondrial respiration compared to their counterparts kept in growth medium at 3 months in culture, as well as compared to differentiated cells throughout the follow-up. Data shown as mean ± SEM (n ≥ 3). (**D**) Maximal mitochondrial respiratory rates show significant increases at months 1–3 in differentiation medium compared to their counterparts kept in growth medium, peaking at month 3. Data shown as mean ± SEM (n ≥ 3). (**E**) Spare respiratory capacity is maintained at elevated levels in differentiated cells compared to their counterparts in growth medium at months 1–3 of the follow-up, peaking at month 3. (**F**) Coupling efficiency reveals significantly increased levels at months 1–3 in culture for differentiated cells compared to controls, maintaining steady levels throughout the follow-up. Data shown as mean ± SEM (n ≥ 3). ** *p* < 0.01 vs. control, *** *p* < 0.001 vs. control; § *p* < 0.05 vs. day 1, §§§ *p* < 0.001 vs. day 1; # *p* < 0.05 vs. 1 month, ### *p* < 0.001 vs. 1 month; $ *p* < 0.05 vs. 2 months, $$$ *p* < 0.001 vs. 2 months.

Measurements of ATP production revealed that differentiated cells rely to a greater extent on aerobic respiration for ATP production, maintaining a steady proportion of approx. 50–70% mitochondrial ATP (mitoATP) out of total ATP production rate, beginning as early as day 1 in culture, while in undifferentiated cells, mitoATP rates drop to approx. 10% with time, relying to a far greater extent on glycolytic ATP (glycoATP) (Figure 8A). While total ATP rates are relatively elevated in controls at day 1, this is reflective of elevated energy demands present in actively dividing cells [14], as opposed to the lower demands characteristic of terminally differentiated postmitotic cells [6,14], which is consistent with previous studies of ARPE-19 behaviour with differentiation [14] and the downregulation of the pro-mitotic apparatus documented upon shifting to low serum-containing media [6,9,69].

The parameters of mitochondrial function were calculated from dynamic oxygen consumption rates (OCR) measured following sequential injection of mitochondrial stressors (Figure 8B). Results showed that basal mitochondrial respiratory rates, reflecting OCR required for routine cell maintenance and survival, are at roughly equal levels in the cells switched to differentiation medium at day 1 compared to controls, consistent with decreased oxygen requirements of postmitotic, serum-deprived cells compared to actively proliferating cells, then begin increasing comparatively to their respective controls at 2 months in culture, reaching their highest level at 3 months in culture, where they surpass both their respective controls, as well as the recorded level at 1 day in differentiation medium (Figure 8C).

Maximal respiratory rates, reflecting OCR achieved when stimuli are applied to force maximal mitochondrial oxidative phosphorylation (OxPhos) activity, followed a consistent pattern to that documented for basal respiratory rates. After similar levels in the differentiation medium-treated cells at day 1 compared to controls, maximal respiratory rate levels were subsequently significantly increased compared to controls at all time points in the follow-up (Figure 8D). Consequently, spare respiratory capacity (SRC), calculated as the difference between maximal and basal respiration, a measure of additional mitochondrial energy expenditure required to meet increased energy demands under conditions of high energy demand/stress, is steadily maintained in the differentiated cells (1–3 months in differentiation medium), while non-differentiated cells kept in growth medium exhibit fluctuating levels, dropping significantly at 1 and 2 months in culture (Figure 8E).

Coupling efficiency, reflecting the effectiveness of mitochondria in converting oxygen consumption into ATP, maintained steady levels under differentiation conditions from day 1 up to 3 months in culture, while substantially dropping in undifferentiated cells kept in growth medium beginning at 1 month in culture (Figure 8F).

Given that pyruvate-containing medium does not bias the system in favour of immediately increasing basal or maximal respiration, as well as the cells’ exposure to constant levels of glucose, it is notable that, following the initial lull at day 1 in respiratory rates owing to serum deprivation, which removes growth signals favouring cell proliferation, serum-free differentiated cultures enter a stage where the RPE differentiation process alone can be seen as the driver behind the steady increase in basal and maximal respiration patterns, to degrees significantly higher than those observed for proliferating cells. This also provides arguments in favour of pyruvate acting independently of its role as a metabolic substrate in order to facilitate differentiation of an RPE phenotype.

Taken together, bioenergetic data reflect that cells kept in differentiation-inducing conditions are more reliant on mitochondrial respiration compared to their undifferentiated counterparts, maintain steady levels of mitochondrial activity, and, while their energetic demands are lower on account of being terminally differentiated, postmitotic cells, they are more efficient at producing mitoATP and better equipped to withstand increased energetic demands and stressful stimuli.

## 4. Discussion

In their original work deriving the ARPE-19 cell line, Dunn et al. [5] characterised it as a spontaneously arising human RPE-derived cell line, possessing the ability to achieve cellular senescence and which, therefore, cannot be considered an immortal cell line [5]. However, further studies assessing ARPE-19 subculture behaviour across a high number of population doublings showed growth patterns suggesting a mix of mortal and immortal characteristics [8]. More recent work has challenged the genetic stability of ARPE-19 cells, documenting abnormalities during metaphases and aneuploidies [64]; this further underscored the dual mortal/immortal subpopulation characteristics of the ARPE-19 cell line [70]. Attempts to isolate the clones with a normal karyotype were unsuccessful, due to the fact that this population would reach senescence [64]. It is, however, important to note that heterogeneity in vitro has been documented for stem cell-derived RPE cultures as well [71,72,73,74], reported to be a concern with regard to their post-transplantation outcomes [74].

It has been posited that while, initially, ARPE-19 exhibited many characteristics of in situ RPE, most have been lost after extensive propagation [9,75] and the line in its naïve state currently exhibits similar features and morphology to fibroblasts and/or mesenchymal stem cells rather than RPE [11]. However, it was found that cells grown under experimental settings of increased confluence differentiated towards an epithelial and, notably, RPE cell state as assessed via transcriptomics [11]. These findings were further corroborated by assessing publicly available transcriptomic data of ARPE-19 from multiple groups, documenting the cell line’s propensity towards epithelial conditioning and differentiation, but also its limited plasticity in terms of further transition towards mesenchymal cell states [11]. While these data are indicative of high plasticity for ARPE-19 cells, they also suggest that their baseline phenotype, at currently available passages, leans towards the mesenchymal end of the epithelial–mesenchymal axis [11].

There is a substantial body of work assessing the transcriptomic relevance of ARPE-19 cells in interrogating fundamental RPE physiological processes, as well as comparability to native RPE tissue. Microarray studies initially showed differences in gene expression between native RPE and ARPE-19 grown in different settings, with confluent, differentiated, and serum-deprived cultures resembling the profile of native RPE the closest [6]. Other microarray studies have also documented differences in gene expression patterns, while highlighting the fact that a considerable number of genes involved in phagocytosis, neurogenesis, and angiogenesis show no differential expression between native RPE and ARPE-19 cells [76]. Later work deployed whole transcriptome RNA-Seq analysis, assessing ARPE-19 in early culture after low-density seeding and after 4 months of differentiation, showing marked upregulation of RPE-specific genes involved in pigmentation, all enzymes of the visual cycle, phagocytosis, cell differentiation, and RPE-related transcription factors [9]. The same study performed whole transcriptome comparison between differentiated ARPE-19, hESC-derived RPE, and hfRPE, on the one hand, and native RPE, on the other, and showed, interestingly, that while there are notable similarities between the in vitro models, all of them exhibit low expression values of crucial RPE genes compared to those in the native RPE [9]; thus, no in vitro model out of the three could be posited to perfectly replicate RPE biology. More recent studies, however, provided further evidence for the usefulness of differentiated ARPE-19, even at 9 days of culture under serum-deprived conditions, for the study of gene expression pattern changes in AMD [69]. In some instances, direct attachment of the cells to the regular tissue culture plastic surfaces was shown to have more relevant outcomes than when coating the surface with protein-based matrix [6]. Another study reported that transcriptomic data for iPSC-RPE showed greater expression of relevant genes compared to ARPE-19 kept in culture for 1 month; however, it is important to note that, in this case, ARPE-19 culture was not serum deprived, having been kept in medium containing 10% FBS which enhances cell proliferation to the detriment of differentiation [77], further underscoring the importance of culture conditions for differentiating physiologically relevant ARPE-19.

It is relevant to note that iPSC-RPE have also shown transcriptional differences compared to native RPE, with genes relating to cell maintenance, retinal development, and muscle contraction significantly upregulated compared to native RPE, resembling that of an immature RPE state [74]. Furthermore, animal studies of implanted human iPSC-RPE subjected to single-cell RNA sequencing showed improved maturation compared to in vitro settings, with gene expression patterns more closely mirroring native adult RPE [72], as well as specific activation of transcription factors *FOS*, *JUND,* and *MAFF* in implanted cells compared to their in vitro counterparts, providing further maturation [74].

Thus, despite their popularity, currently commercially available ARPE-19 cells provided at relatively high passages present a number of limitations, including gradual reduction in viability and telomere length [8], rapid senescence, heterogenous karyology and chromosomal abnormalities [10,13,69], an unrepresentative transcriptome and putative insufficient degree of differentiation [6,75], as well as reported inconsistency among groups [10]. An additional area of concern is the improper use of the cell line, with a large number of studies employing ARPE-19 for experiments at non-confluent cell densities, in an undifferentiated state, and using regular, high serum-containing growth culture medium.

Subsequent studies have evaluated these limitations, aiming either to refine and improve currently existing protocols to ensure physiological relevance of ARPE-19-based studies, or to make the best use of the advantages the cell line offers. Hazim et al. [13], despite corroborating previously attested chromosomal abnormalities [69] in passage 17 ARPE-19 cells, concluded that the presence of these aberrations does not affect the cells’ ability to differentiate into an RPE-like state [13]. Low passage cell cultures have been proposed as a solution to mitigate and/or circumvent these potential shortcomings, as they exhibit more of the native RPE features and are more prone to maturing to a differentiated phenotype under appropriate culture conditions [9]. Good practices for continued and consistently reproducible work employing ARPE-19 have been put forth [10]. These include detailing cell and reagent origin, providing imaging documenting a differentiated state with regard to morphology and pigmentation, confluence, panels of RPE signature genes and proteins, as well as functional assays demonstrating polarisation [10].

Previous efforts have been made to create appropriate culture conditions for ARPE-19 cells to achieve a suitably differentiated, physiologically relevant phenotype in vitro [7,9,13,14,15,16], highlighting the importance of factors such as a high glucose-containing medium [7], pyruvate [7,9], nicotinamide supplementation [13,14,15,16], the use of low passage cell stocks [9], and culture on ECM-coated transwells to simulate in situ polarised conditions [7,9,13,14,16]. Nicotinamide, in particular, has been used for the differentiation of iPSC-RPE [78,79], retinal organoids [80], and improving disease markers in RPE models of AMD [81,82]. It was also proposed as a facilitator of rapid ARPE-19 differentiation in addition to standard media formulations [13,14,16]. The initial reasoning behind this was that a large number of nicotinamide adenine dinucleotide (NAD+)-dependent enzymes are involved in cellular respiration-related redox reactions during the Krebs cycle and oxidative phosphorylation (OxPhos) and in biosynthetic reactions such as the processing of DNA, RNA, proteins, and secondary messengers cADP-ribose and NAADP+ [83]. Nicotinamide is a crucial precursor of NAD+; its supplementation in cell culture results in increased NAD+ levels [84]. This increase consequently results in upregulation of mitochondrial genes [14,84,85] and protein expression, as well as relevant changes in mitochondrial morphology and size [14]. Moreover, a short-term ARPE-19 differentiation study showed that nicotinamide also enhances other RPE-related phenotypical traits, notably, viral tropism of AAV1 towards the cells, at levels comparable to the tropism of AAV1 towards native RPE [15].

Closer dissection of mechanisms behind nicotinamide-mediated ARPE-19 differentiation towards an RPE phenotype outlined the role of miR-494-3p repression, consequently increasing the ratio of PTEN/SIRT3 with a stabilising role on mitochondrial homeostasis [86,87]. Furthermore, nicotinamide acts via the induction of SIRT1 [82,88], a NAD-dependent deacetylase with protective roles against stressors [89] widely considered to exert protective effects against cellular senescence and ageing [90]. Thus, despite exerting substantial effects on metabolism, nicotinamide is currently not considered to have direct effects on altering the expression of genes involved in RPE maturation and functions [81,82]. NAD deficiency has, additionally, been noted to occur early in the onset of ocular disorders, including diabetic neuropathy and light-induced and age-dependent photoreceptor degeneration [82,91], and has been shown to curb them when supplemented by mitigating the accumulation of mitochondrial dysfunction and defects [82,92,93,94].

While the precise means through which pyruvate acts to facilitate differentiation of ARPE-19 in long-term culture has not been definitively identified [7], it has been shown that pyruvate independently improves pigmentation, CRALBP, and MerTK expression [7]. It has been suggested that the mechanism through which it achieves these effects is independent of its role as an energy substrate, but rather by acting as a scavenger for hydrogen peroxide and other ROS [95,96,97]. Sodium pyruvate has been shown experimentally to possess antioxidant and anti-inflammatory effects, preventing ROS generation and protecting against mitochondrial toxicity [98]. Furthermore, pyruvate was shown to facilitate, and subsequently routinely used for, the improvement of in vitro and in vivo differentiation in other cell types as well, including adipocytes [99], skeletal muscle [100], hematopoietic stem cell fate specification [101], and neuronal stem cells [102]. Crucially, pyruvate has been shown to lie at the point of integration between the metabolome and epigenome [103], controlling chromatin remodelling by means of histone acetylation [103,104,105] and lactylation [105] and, consequently, integrating key signalling pathways such as Wnt, PI3K-AKT, JAK-STAT, and Hippo [106].

The present work built upon these prior findings, aiming to put forth good practices in terms of achieving RPE phenotype for physiologically relevant modelling using ARPE-19 cells, and most importantly, ascertaining the temporal relationship of the acquisition of RPE features, including minimal and optimal thresholds for use in physiologically relevant modelling. The findings provide ample characterisation of the differentiation process, indicating critical points at which specific metabolic, alongside morphologic and gene expression characteristics, become significant and meaningful for appropriate interpretations.

Remarkably, a plethora of RPE features were achieved early in culture, i.e., at 1 month or even earlier; this is the case of TER achieving polarisation, peak gene and protein expression for tight junction component OCLN, core membrane transporters such as BEST1, key visual cycle enzymes RPE65 and CRALBP, notably all assayed mitochondrial fusion and fission regulators, expansion of mitochondrial mass, potential, and network. Increases in mitochondrial area, branch number, and branch length were detected as early as 2 weeks in culture, indicating rapidly shifting metabolic changes under appropriate growth conditions.

Some RPE markers required longer periods of maturation to reach their maximum values, notably, melanin accumulation, beginning with 3 months in culture, mitochondrial area, branch number and length at 2 months, and basal respiratory rate at 3 months in culture. However, a remarkable and unexpected finding was that, in most cases, significant increases of these parameters were detected at 1 month in culture (pigmentation, TYR gene and protein expression) and even earlier, as was the case for mitochondrial area and branching, underscoring the fact that low passage ARPE-19 cells at 1 month in differentiation-promoting culture conditions fulfil critical relevant criteria for conducting physiologically relevant in vitro studies. Given the temporal differences in achieving peak expression for some functional RPE features, it can be inferred that there is a temporally controlled and hierarchical activation [107,108,109,110,111] and differentiation of RPE phenotypic markers in long-term culture of ARPE-19 cells kept under low-serum, high-glucose, pyruvate- and nicotinamide-supplemented culture medium.

While previous works stressed long-term cultures to achieve similar outcomes, recommending protocols as long as 3 [7] or even 4 months [9], we show here that further nicotinamide supplementation dramatically shortens the necessary duration of the protocol required to achieve sufficient differentiation in order to study most key RPE functions, while also achieving TYR overexpression and pigmentation at 1 month in culture, which currently available short-term protocols have not fully achieved [13,14,16]. Such alternative short-term protocols are, nonetheless, valuable when conducting studies where polarity and RPE marker expression in the absence of pigmentation constitute an advantage, such as fluorescence-based assays [13], or instances in which high glucose medium may be a hindrance to studying certain metabolic pathways [16].

The energy metabolism of the visual system, notably that of the RPE and neuroretina, is a complex topic. Current models suggest that photoreceptor cells in the neuroretina exhibit a Warburg-like metabolism, relying on aerobic glycolysis, whereby blood glucose supplied by the choriocapillaris via the RPE is converted by the photoreceptor cells into lactate, at least most of which is subsequently shuttled to RPE cells where oxidative phosphorylation occurs, fuelling their own metabolic needs [19]. To achieve this, glucose consumption in the RPE is suppressed, increasing the amount of glucose that can be delivered to the photoreceptor outer segments [19]. It is in light of these peculiarities that the value of the current model is to be understood. While the relatively elevated levels of ATP production rates and OCR in the controls (undifferentiated cells in growth medium, day 1 in culture) seem to suggest higher metabolic activity, it is notable that these are dividing cells that, even when confluent, tend to grow atop one another in both short- and long-term cultures [14]. Conversely, cells kept in physiological phenotype-inducing conditions are postmitotic, terminally differentiated and, consequently, their metabolic requirements are, on the whole, lower compared to actively dividing cells. Notably, these are cells which are heavily reliant on oxidative phosphorylation, where the bulk of their energy needs are covered by mitoATP and where spare respiratory capacity and coupling efficiency are elevated. The findings are consistent with previous work aiming at investigating cellular respiration under differentiation conditions [14,16], while maintaining the cells in culture for a substantially longer follow-up and documenting the timeline of the maintenance of a steady state for the differentiated cells up to and including 3 months in culture. While comparing proliferating to non-proliferating cells can be seen as a potential confounder for the experiment, glucose handling by terminally differentiated cells vs. proliferating cells has been amply discussed and documented [112]. Furthermore, pyruvate was previously documented to increase the rate of basal respiration, suggestive of rate-controlling effects in some cell types, both primary and immortalised [113]; therefore, it was crucially important to assess whether pyruvate, a long-standing staple supplement of ARPE-19 differentiation media [7,9,13,16], would thus bias the present model. The documented initial lull in basal respiration following the switch to pyruvate-containing media suggests that this is not the case, with the interplay of proliferation/terminal differentiation being of greater import in this scenario.

## 5. Conclusions

**Figure 9:**
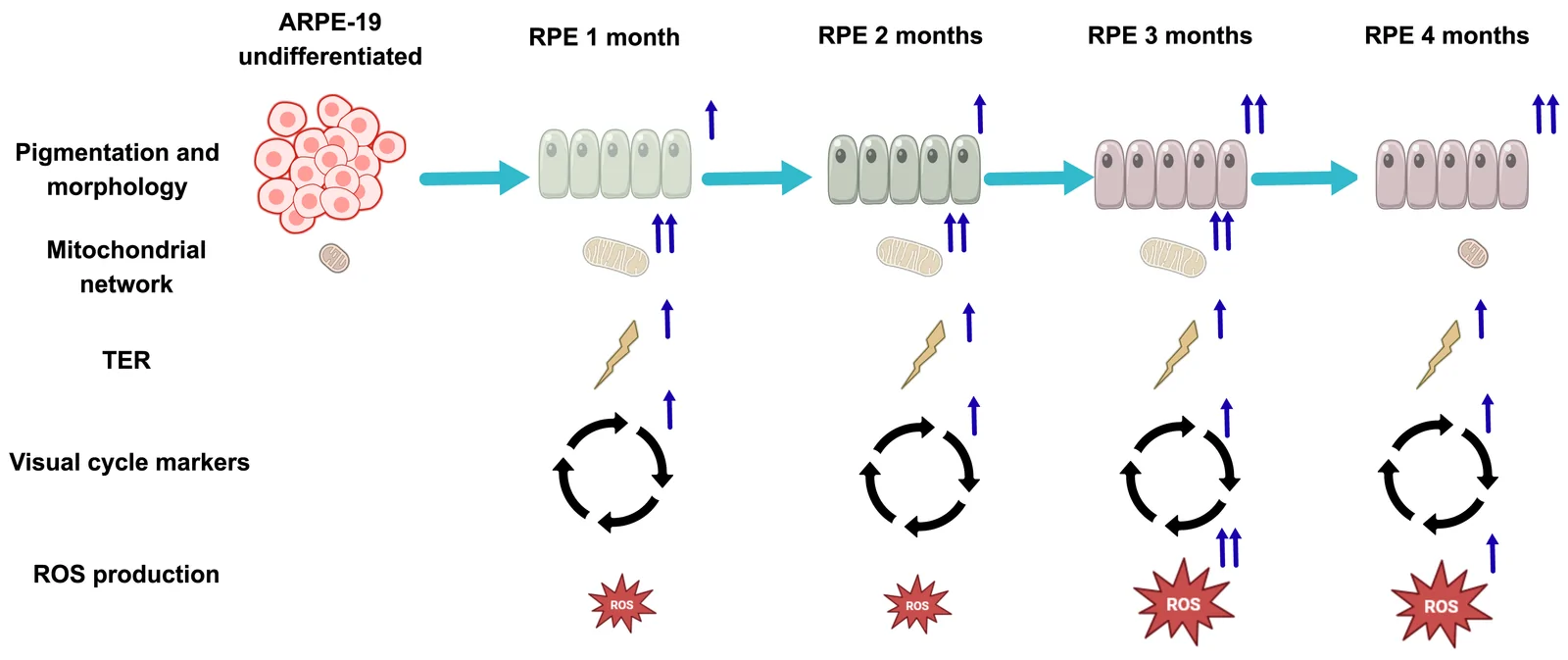
Graphic summary of the findings. ARPE-19 differentiated under physiological phenotype-inducing conditions achieve increased mitochondrial mass/volume and activity, polarity as measured by TER, and visual cycle component expression early on during the follow-up, exhibiting features of functional maturity as early as 1 month in culture. With the exception of mitochondrial mass, which shows a relative decline around 4 months in culture, all other characteristics are maintained at relatively constant levels. This decline is accompanied by increases in ROS production late during the follow-up, notably at 3 and 4 months in culture. Melanin accumulation increases significantly starting at 1 month in long-term culture, peaking and plateauing at 3 months. Taken together, the data support the feasibility of early usage of long-term differentiated low-passage ARPE-19 for RPE functional studies.

In conclusion, ARPE-19 are a useful and accessible tool for the study of RPE, but require differentiation/maturation in order to be physiologically relevant. In the present work, we have analysed polarisation, pigmentation, functional markers, mitochondrial dynamics, and metabolism in long-term culture, showing that a combination of low-passage cells, differentiation media supplemented with pyruvate and nicotinamide, as well as BrM-like ECM are crucial to producing a phenotype where most, if not all, crucial functions of the RPE can be assessed. Moreover, a 1-month differentiation protocol based on the principles outlined herein, with due verification of relevant functional and structural markers, is sufficient, if not optimal, to cover the study of most relevant cellular processes in the RPE (Figure 9).

## Data Availability

All data used in the studies are presented here.

## Supplementary Materials

The following supporting information can be downloaded at: https://www.mdpi.com/article/10.3390/cells15201846/s1, Table S1: qRT-PCR primers for gene expression studies. Refs. [114,115,116,117,118,119,120,121,122,123,124] are cited in the Supplementary Materials.
