# Supplementary material for: Timeline of Achieving a Differentiated RPE Phenotype and Specialised Metabolism of ARPE-19 Cells Under Physiologically Relevant-Inducing Culture Conditions
Source: Cells. 2026 Oct 12;15(20):1846. doi: 10.3390/cells15201846 (PMC13702982; doi:10.3390/cells15201846)
Supplement: Supplementary file 1 [file cells-15-01846-s001.zip › cells-4325260-supplementary.pdf]

Table S1. qRT-PCR primers for gene expression studies

Gene	Forward primer	Reverse primer	Reference
TYR	5′TAGCGGATGCCTCTCAAAGC3′	5′CAATGGGTGCATTGGCTTCT3′	[114]
OCLN	5′GGAGGACTGGATCAGGGAAT3′	5′TCAGCAGCAGCCATGTACTC3′	[13]
TJP1	5′AGCCATTCCCGAAGGAGTTGAG3′	5′ATCACAGTGTGGTAAGCGCAGC3′	[115]
BEST1	5′TGCCAACCTGTCAATGAAGGCG3′	5′TCCAGTCGTAGGCATACAGGTG3′	[116]
RPE65 exon 2	5′GGAGTGAACAGGCTTTGAGC3′	5′AAACCACCTGATCCCTCTCC3′	[47]
RPE65 exon 9-11	5′GATCTCTGCTGCTGGAAAGG3′	5′TGGGGAGCGTGACTAAATTC3′	[13]
RPE65 exon 10-11	5′GCTCCCCAACCTGAAGTTAG3′	5′GATAGTCTCGTCACTGCACAG3′	[16]
RDH5	5′CTGTGACCAACCTGGAGAGTCT3′	5′GATGCGCTGTTGCATTTTCAGGT3′	[117]
RBLP1	5′CCTACAATGTGGTCAAGCCCTTC3′	5′CGAAGTCAGAGGGCAGGATGTT3′	[118]
MFN1	5′GGTGAATGAGCGGCTTTCCAAG3′	5′TCCTCCACCAAGAAATGCAGGC3′	[119]
MFN2	5′CTCTCGATGCAACTCTATCGTC3′	5′TCCTGTACGTGTCTTCAAGGAA3′	[120]
OPA1	5′GTGGTTGGAGATCAGAGTGCTG3′	5′GAGGACCTTCACTCAGAGTCAC3′	[121]
DRP1	5′GATGCCATAGTTGAAGTGGTGAC3′	5′CCACAAGCATCAGCAAAGTCTGG3′	[122]
FIS1	5′ATCCGTAAAGGCATCGTGCT3′	5′TCGTATTCCTTGAGCCGGTAG3′	[123]
GAPDH	5′ACCACAGTCCATGCCATCAC3′	5′TCCACCACCCTGTTGCTGTA3′	[124]
